# Triterpenoid and Steroid Content of Lipophilic Extracts of Selected Medicinal Plants of the Mediterranean Region

**DOI:** 10.3390/molecules28020697

**Published:** 2023-01-10

**Authors:** Leila Gadouche, Abdulwadood Shakir Mahmood Alsoufi, Dominika Pacholska, Anna Skotarek, Cezary Pączkowski, Anna Szakiel

**Affiliations:** 1 Department of Biology and Physiology of Organisms, Faculty of Biological Sciences, University of Sciences and Technology Houari Boumediene, P.O. Box 32, El Alia, Bab Ezzouar, Algiers 16111, Algeria; 2 Laboratory of Natural Bio-Resources, Faculty of Natural and Life Sciences, Hassiba Benbouali University of Chlef, P.O. Box 151, Chlef 02000, Algeria; 3 Department of Biology, College of Science, University of Tikrit, P.O. Box 42, Tikrit 34001, Iraq; 4 Department of Plant Biochemistry, Faculty of Biology, University of Warsaw, 1 Miecznikowa Street, 02-096 Warsaw, Poland

**Keywords:** *Cistus ladanifer*, *Cistus monspeliensis*, *Erica arborea*, *Globularia alypum*, *Pistacia lentiscus*, *Rhamnus alaternus*, steroids, triterpenoids

## Abstract

The available phytochemical characteristics of the medicinal plants and derived herbal material often lack data concerning the content of steroids (including phytosterols) and triterpenoids, that can be responsible for various beneficial properties and therapeutic effects, either directly, or as a result of synergistic action with other bioactive constituents. The aim of the present work was the analysis of the content of these compounds in herbal material (leaves, aerial parts) derived from selected medicinal plants (*Cistus ladanifer*, *Cistus monspeliensis*, *Erica arborea*, *Globularia alypum*, *Pistacia lentiscus*, *Rhamnus alaternus*), widely used in folk medicine in the Mediterranean region. Results obtained by gas chromatography-mass spectrometry (GC-MS)-targeted profiling revealed the diversity in the profiles and contents of steroids and triterpenoids in the analyzed plant material, ranging from 5.7% d.w. in *E. arborea* to 0.1% in *G. alypum*. The obtained results supplement the existing phytochemical data of the investigated medicinal plants, pointing to the *E. arborea* aerial parts and *P. lentiscus* leaves as valuable resources of phytosterols and bioactive triterpenoids.

## 1. Introduction

Medicinal plants have been used in traditional medicine systems for thousands of years [1,2]. In last decades, the use of herbal remedies has acquired a renewed interest, and they are often considered as dietary supplements for disease prevention, as well as the alternative or complementary treatment for various ailments, particularly lifestyle diseases [3]. Medicinal plants also serve as the potential source of new therapeutic agents, or prototypes for the development of new synthetic or semisynthetic drugs [4]. 

The available phytochemical characteristics of the medicinal plants and derived herbal material often lack data concerning the content of two specific groups of isoprenoid compounds, i.e., steroids (including phytosterols) and triterpenoids. These compounds, although usually present in plant material in relatively low amounts, exert important biological activities and can be responsible for some pharmacological properties, either directly, or as a result of synergistic action with other bioactive constituents, e.g., polyphenols [5,6]. Phytosterols reduce lipid and cholesterol plasma levels, including low-density lipoprotein cholesterol (LDL-C), and may have clinical application for the prevention of cardiovascular diseases, as well as fatty liver, inflammatory, rheumatoid arthritis and obesity-related diseases. Phytosterols also play other roles in promoting human health, e.g., improving insulin resistance and lipid metabolism [7,8,9]. Triterpenoids, owing to their structural diversity, exert an enormous range of bioactivities, including anti-inflammatory, antimicrobial, antiviral, hepatoprotective, antidiabetic and anticarcinogenic properties, which have significant pharmaceutical and industrial applications [10,11,12,13]. Recently, triterpenoids have received much attention due to the possibility of their application as novel therapeutic agents against multidrug-resistant microbial and fungal strains [14,15].

The aim of the present work was the analysis of the content of these two groups of important phytochemicals in herbal material (leaves, aerial parts) derived from selected medicinal plants (*Cistus ladanifer*, *Cistus monspeliensis*, *Erica arborea*, *Globularia alypum, Pistacia lentiscus*, *Rhamnus alaternus*), widely used in folk medicine of various human populations in the Mediterranean region. Due to the unique geographical situation, unrivaled environmental heterogeneity and specific climate, the Mediterranean region has a very diversified floristic richness, considered “a global hotspot of endemic vascular plants”. Historical development of diverse civilizations resulted also in particularly broad traditional knowledge of medicinal and aromatic species [16]. The plants selected for the present study were collected in Beni Haoua in Algeria, a region known for the particular floral diversity. The investigated plant parts have been widely utilized in traditional medicine by autochthon populations.

*Cistus* L. (rockrose) is a genus indigenous to the Mediterranean region, comprising dicotyledonous perennial herbaceous plants, traditionally used in folk medicine as herbal tea infusions for healing digestive problems and colds, as extracts for the treatment of various diseases, and as fragrances [17]. Extracts obtained from dry leaves of *C. ladanifer* L. are used for their antibacterial, antifungal, antispasmodic and antioxidant activities; for the treatment of diarrhea, various skin diseases, and as anti-inflammatory agents [18]. Extracts from *C. monspeliensis* L. also exert antimicrobial, antioxidant, and anti-inflammatory properties; they are applied against hyperglycemia and diabetes [19,20].

*Erica* species (Ericaceae) are used in folk medicine for their therapeutic properties including antimicrobial, antiviral, diuretic, anti-inflammatory, antinociceptive and antiulcer activities [21]. *Erica arborea* L. (tree heath) is an evergreen shrub or small tree, native to south-west Europe, the Mediterranean region and northern Africa. Extracts from leaves and flowers of *E. arborea* L. are used as antirheumatic, diuretic and astringent agents, and in the treatment of urinary tract infections [22,23].

*Globularia* L. genus consists of herbs, chamaephytes or shrubs, common in the Mediterranean region. *G. alypum* (known under many local names as Tasselgha, zriga, Ain Larneb) is widely used in folk medicine for anti-inflammatory, antiulcer, antioxidant properties; in the treatment of cardiovascular and renal diseases, and various cancer lesions of the stomach, colon, rectum and oesophagus [24,25]. In North African countries, it is one of the most frequently cited plant species used for diabetes [26].

*Pistacia* L. genus (Anacardiaceae) comprises evergreen or deciduous resin-bearing shrubs and trees. *P. lentiscus* is most commonly used in different regions as a therapeutic agent in the treatment of digestive, hepatic, and kidney diseases. Extracts of different parts of the plant exert various activities, such as antioxidant, anti-inflammatory, antiproliferative, and neuroprotective effects [27,28]. 

*Rhamnus alaternus* L. (Rhamnaceae) is a small shrub (known under many local names in North Africa, e.g., Imlilesse, Oud El-khir, Safir; or Meliles in Berber language), used in traditional medicine for its gastric, diuretic, hepatoprotective, hypotensive, antioxidant antimutagenic effects. Extracts from various parts of *R. alaternus* (i.e., roots, bark, berries, leaves) are applied to treat a large number of disorders, including diabetes, hepatitis, dermatological and goiter problems [29,30].

The targeted profiling of steroids and triterpenoids by gas chromatography-mass spectrometry (GC-MS) method performed in this study complements the available phytochemical characteristics of the selected plants, and indicates the promising herbal sources of the analyzed compounds.

## 2. Results

### 2.1. Identification of Steroids and Triterpenoids in Obtained Extracts

All compounds were analyzed by the gas chromatography-mass spectrometry (GC-MS) method and identified according to their MS spectra (steroids and neutral triterpenoids without derivatization, triterpenoid acids after methylation); the identification was additionally supported by comparison of their retention time and chromatographic mobility to the respective parameters of available authentic standards, as well as comparison with data from MS libraries and literature (Section 4.5, Table S1).

**Figure 1:**
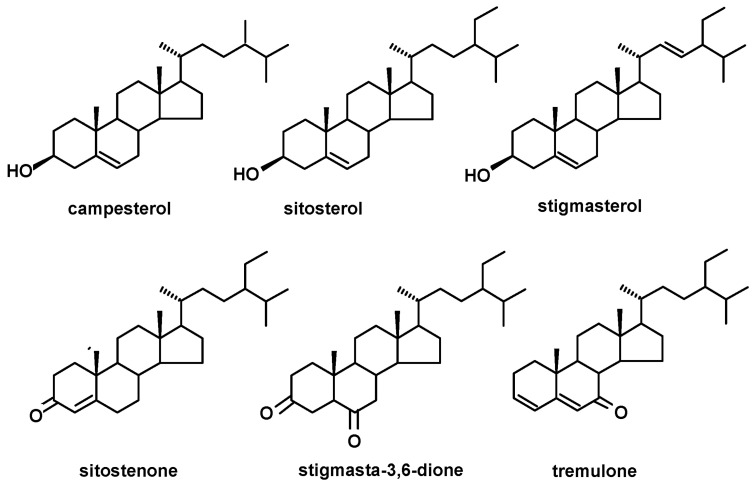
The structures of identified phytosterols and steroids.

GC-MS analysis of the fractions containing steroids and neutral triterpenoids obtained from diethyl ether extracts of analyzed plants revealed the presence of three typical plant sterols, belonging to the group commonly known as phytosterols, i.e., campesterol [(24*R*)-ergost-5-en-3β-ol], sitosterol [stigmast-5-en-3β-ol] and stigmasterol [(22*E*)-stigmasta-5,22-dien-3β-ol]. The oxygenated derivatives of sterols (steroid ketones): sitostenone (stigmasta-4-en-3-one), stigmasta-3,6-dione and tremulone (stigmasta-3,5-dien-7-one) were also identified. The structures of the identified steroids are presented in Figure 1. 

**Figure 2:**
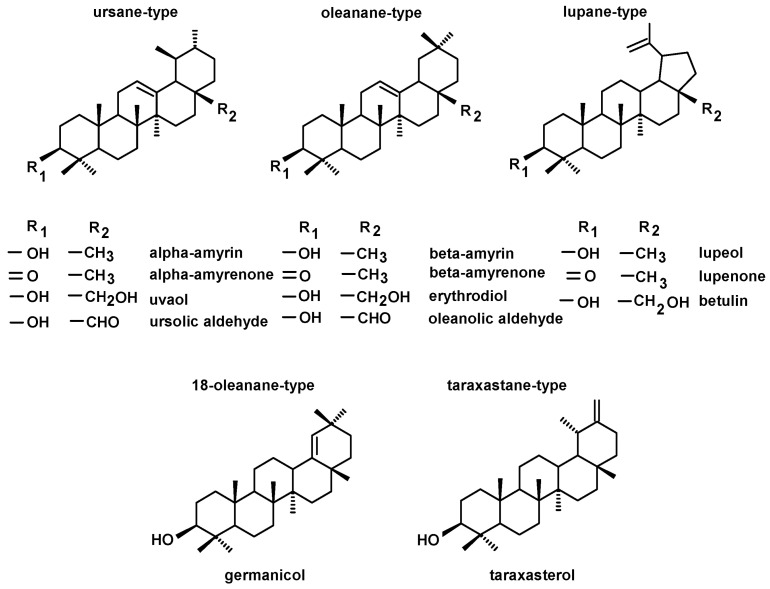
The structures of identified neutral triterpenoids.

Among the neutral triterpenoids, the two most commonly occurring triterpenoid alcohols with one hydroxyl group (monols) of ursane- and oleanane-type compounds were found, i.e., α-amyrin (urs-12-en-3β-ol), and β-amyrin (olean-12-en-3β-ol). Other identified monols were belonging to lupane-type triterpenoids (lupeol, lup-20(29)-en- 3β-ol), taraxastane-type triterpenoids (taraxasterol, taraxast-20(30)-en-3β-ol) and 18-oleanane-type (germanicol, olean-18-en-3β-ol). Oleanane-, ursane- and lupane-type alcohols with two hydroxyl groups (diols) comprised erythrodiol (olean-12-ene-3β,28- diol), uvaol (urs-12-ene-3β,28-diol), and betulin (lup-20(29)-ene-3β,28- diol). Ketones, i.e., α-amyrenone, β-amyrenone and lupenone, as well as oleanolic and ursolic aldehydes, were also identified. The two identified monols, α-amyrin and lupeol, and their ketones, α-amyrenone and lupenone were associated with common peaks, as described in the previous reports [31,32]; therefore, in the extracts where lupane-type triterpenoids were present, the respective pairs of compounds were quantified together. Some triterpenoid esters, i.e., lupeol acetate and maslinic acid methyl ester, were identified in the fraction of the neutral triterpenoids, due to the same range of their chromatographic mobility during fractionation on silica gel plates (Rf 0.3–0.9; Section 4.3). The calculated content of maslinic acid methyl ester was then included in the fraction of triterpenoid acids. The structures of the identified neutral triterpenoids, classified according to the skeleton type, are presented in Figure 2.

**Figure 3:**
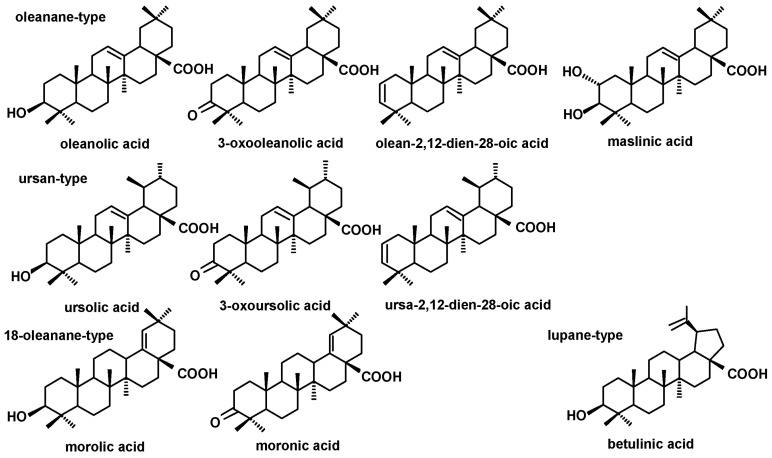
The structures of identified triterpenoid acids.

In the fractions of triterpenoid acids (subjected to methylation prior to GC-MS analysis) also the groups of ursane- and oleanane-type compounds were identified, comprising ursolic acid (3β-hydroxy-urs-12-en-28-oic acid) and oleanolic acid (3β-hydroxy-olean-12-en-28-oic acid), accompanied by their various derivatives: 3-oxo-analogs (3-oxoolean-12-en-28-oic acid and 3-oxours-12-en-28-oic acid), analogs with additional double bond in position 2 (olean-2,12-dien-28-oic acid and ursa-2,12-dien-28-oic acid), and 2,3-dihydroxy analog of oleanolic acid (2α,3β-dihydroxy-olean-12-en-28-oic, i.e., maslinic acid). One acid belonging to lupane-type triterpenoids, i.e., betulinic acid (3β-hydroxy-lup-20(29)-en-28-oic acid) and two 18-oleanane-type acids (morolic acid, 3β-hydroxy-olean-18-en-28-oic acid and moronic acid, 3-oxoolean-18-en-28-oic acid) were also identified. The structures of the identified triterpenoid acids are presented in Figure 3. 

### 2.2. The Content of Steroids and Triterpenoids in Cistus ladanifer and Cistus monspeliensis

The steroid and triterpenoid profiles of analyzed leaf extracts of *C. ladanifer* and *C. monspeliensis* were similar. They contained the most typical phytosterols, i.e., campesterol, stigmasterol and sitosterol, accompanied by steroid ketones: sitostenone, stigmastan-3,6-dione and tremulone. The fraction of the neutral triterpenoids consisted only of α- and β-amyrins. In the fraction of the triterpenoid acids, ursolic and oleanolic acids were identified, along with 3-oxo-oleanolic acid and maslinic acid. In *C. monspeliensis* leaf extract, additionally the naturally occurring methyl ester of maslinic acid was identified in the fraction of the neutral triterpenoids. 

The results of the quantitative analysis of identified compounds are presented in Table 1.

The total content of identified steroids and triterpenoids constituted approx. 0.13% of *C. ladanifer* leaf dry weight, and 0.2% of leaf d.w. in *C. monspeliensis*. Phytosterols were the most abundant fraction in *C. ladanifer* (approx. 50% of the total content of all identified compounds), whereas the neutral triterpenoids were the predominating fraction (39%) in *C. monspeliensis*. 

Among phytosterols, sitosterol was the most abundant (78% of the fraction) in *C. ladanifer* leaves, α-amyrin among the neutral triterpenoids (70% of the fraction), and ursolic acid among triterpenoid acids (41%). Similarly, sitosterol was the most abundant phytosterol (74% of the fraction) in *C. monspeliensis* leaves, and α-amyrin among the neutral triterpenoids (57% of the fraction). However, maslinic acid was predominating (59%) among triterpenoid acids in *C. monspeliensis*, while ursolic acid was the less abundant. 

Apart from steroids and triterpenoids, the GC-MS analysis of the diethyl ether extract obtained from *C. ladanifer* leaves revealed the occurrence of some other characteristic isoprenoids, e.g., campherene C_15_H_24_O and cyperene C_15_H_24_, being volatile sesquiterpenoids; and diterpenoid phytol (C_20_H_40_O). In turn, aliphatic alcohol β-citronellol (C_10_H_20_O), and known antioxidants, α- and β-tocopherols (C_29_H_50_O_2_), were identified in *C. monspeliensis* leaf extract. 

### 2.3. The Content of Steroids and Triterpenoids in Erica arborea

The results of the quantitative analysis of compounds identified in the aerial part (the mixture of flowers and leaves of *E. arborea* are presented in Table 2. 

In the extracts obtained from the *E. arborea* aerial part, being the mixture of flowers and leaves, two phytosterols, i.e., campesterol and sitosterol, and two steroid ketones, sitostenone and tremulone, were identified. In turn, the profile of triterpenoids was very complex. The fraction of the neutral triterpenoids consisted of alcohols with one hydroxyl group (both amyrins, lupeol accompanied with its acetate, and taraxasterol); alcohols with two hydroxyl groups (erythrodiol, uvaol, and betulin); ketones (α-amyrenone, β-amyrenone, lupenone) and oleanolic and ursolic aldehydes. In the fraction of triterpenoid acids, betulinic, oleanolic and ursolic acids were identified, along with olean-2,12-dien-28-oic and ursa-2,12-dien-28-oic acids, 3-oxo-oleanolic and 3-oxo-ursolic acids, and maslinic acid

The obtained results revealed that the total content of steroids and triterpenoids reached almost 5.7% of the *E. arborea* aerial part dry weight, making this plant material the very abundant source of these compounds. The contents of the fraction of the neutral triterpenoids and triterpenoid acids were comparable, constituting 2.8% and 2.7% d.w., respectively. Among the neutral triterpenoids, the most predominant compounds were α-amyrin and lupeol (83% of the fraction), followed by β-amyrin (8%). The most abundant triterpenoid acid was ursolic acid (55% of the fraction), the second abundant was oleanolic acid (22%). Phytosterols constituted approx. 0.1 % d.w., with predominating sitosterol (74% of the phytosterol fraction). 

### 2.4. The Content of Steroids and Triterpenoids in Globularia alypum Leaves

Extract obtained from *G. alypum* contained typical phytosterols, i.e., campesterol, sitosterol and stigmasterol; and steroid ketones: sitostenone, stigmastane-3,6-dione and tremulone. Triterpenoids were represented by α- and β-amyrins, lupeol, α-amyrenone and lupenone, betulin, as well as oleanolic and ursolic acids. The total content of both groups of analyzed compounds constituted approx. 0.1% of leaf d.w. Phytosterols were the main constituents (almost 0.05% d.m.) with dominating sitosterol (91% of the sterol fraction). Among triterpenoids, the most abundant compound was ursolic acid (24% of the total triterpenoid fraction), whereas the second most abundant was betulin (23%). 

The GC-MS analysis of the diethyl ether extracts obtained from *G. alypum* leaves revealed additionally the occurrence of numerous long-chain fatty alcohols, including policosanols, e.g., docosan-1-ol (C_22_H_46_O), tetracosan-1-ol (C_24_H_50_O) and octacosan-1-ol (C_28_H_58_O). Moreover, significant peaks of phytol, α-tocopherol (C_29_H_50_O_2_) and α-tocopherylchinon (C_29_H_50_O_2_) were also noticed. 

The results of the quantitative analysis of the steroids and triterpenoids identified in *G. alypum* leaves are presented in Table 3. 

### 2.5. The Content of Steroids and Triterpenoids in Pistacia lentiscus Leaves

Typical phytosterols, i.e., campesterol, sitosterol and stigmasterol, accompanied by two ketones, sitostenone and tremulone, were identified in the extracts from *P. lentiscus* leaves. The composition of neutral triterpenoids was relatively complex, it included alcohols with one hydroxyl group (both amyrins and lupeol) or two hydroxyl groups (erythrodiol, uvaol and betulin), ketones (α-amyrenone and lupenone), as well as oleanolic and ursolic aldehydes. In turn, the fraction of triterpenoid acids was composed of oleanolic acid, 3-oxooleanolic acid, but also moronic acid and morolic acid. 

The obtained results pointed to *P. lentiscus* leaves as an abundant source of both steroids and triterpenoids, the total content of both groups of analyzed compounds reached almost 1.2% d.w. Triterpenoids constituted the prevailing fraction (above 1% d.w.) The neutral triterpenoids (constituting 61% of all triterpenoid compounds) were more abundant than the fraction of acids. The mixture of α-amyrin and lupeol was prevailing among the neutral triterpenoids (81% of this fraction), whereas 3-oxo-oleanolic acid was dominating among acids (constituting 81% of the fraction of acids). Among phytosterols, again sitosterol was the most abundant (95%).

Additionally, phytol, α-tocopherol and α-tocopherylchinon were found in the *P. lentiscus* leaf extract. Moreover, one sesquiterpenoid, β-caryophyllene oxide (C_15_H_24_O), and diterpenoid sclareol (lambd-14-ene-8,13-diol, C_20_H_36_O_2_) were also identified.

The results of the quantitative analysis of the steroids and triterpenoids identified in *P. lentiscus* leaves are presented in Table 4. 

### 2.6. The Content of Steroids and Triterpenoids in Rhamnus alaternus Leaves

The profile of phytosterols in diethyl ether extract obtained from *R. alaternus* was typical, comprising campesterol, sitosterol and stigmasterol, accompanied by three steroid ketones: tremulone, stigmastane-3,6-dione and sitostenone. The composition of triterpenoids was rather simple, since only alcohols: both amyrins and germanicol; as well as two aldehydes (oleanolic and ursolic) were identified. The content of the identified compounds is presented in Table 5.

The total content of steroids and triterpenoids was approx. 0.2% of leaf d.w., with triterpenoids as the dominating class (74% of all identified compounds). Triterpenoid alcohols were the most abundant, with predominating α-amyrin (constituting 32% of triterpenoid fraction), followed by β-amyrin (25%) and germanicol (23%). In the fraction of triterpenoid acids, only traces of oleanolic and ursolic acids were found, below the limit of quantification. Steroids and phytosterols constituted the minor class of the identified compounds, with dominating sitosterol (71% of phytosterol fraction).

### 2.7. Determination of Radical Scavenging Activity of Extracts

The antioxidant potency of analyzed extracts was evaluated using the DPPH (2,2-diphenyl-1-picrylhydrazyl) method to measure free radical scavenging ability. The Radical Scavenging Activity was calculated, as described in Section 4.6, at the highest concentration tested, i.e., 500 µg/mL. The results are presented in Table 6. 

The highest antioxidant potential was exerted by *G. alypum* extract, whereas the lowest (three-fold less) was noticed for *C. ladanifer* extract.

## 3. Discussion

The results obtained in this study presented the diversity in the profiles and contents of steroids and triterpenoids in the analyzed plant material. Aerial parts (the mixture of flowers and leaves) of *E. arborea* and leaves of *P. lentiscus* were the most abundant sources of these compounds, reaching 5.7% and 1.2% d.w, respectively. However, although the contents of steroids and triterpenoids in leaves of other plants were not particularly high (ranging from 0.1% d.w. in *G. alypum* to 0.22% in *R. alaternus*), the gathered data on their composition can valuably complement the available phytochemical characteristics of the analyzed herbal materials and provide new insights into their potential bioactivity.

The therapeutic properties of the extracts obtained from the medicinal plants obviously depend not only on the composition of the main fractions of bioactive constituents, but also on the synergistic effects exerted by their mixtures, even if individual compounds are not present in prevailing amounts. Combination of bioactive compounds with different mechanisms of action seems to be particularly promising due to the increased efficacy and extended spectrum of effects. Such synergism of triterpenoids and phenolic compounds has been reported for antioxidant [33] and antimicrobial properties [5,6]. Specifically, the antioxidant potential was increased due to the cooccurrence of quinonemethide triterpenes and flavonoids in the root bark of *Maytenus ilicifolia* Mart. ex Reissek (Celastraceae), a native Tropical Atlantic Forest plant widely used in traditional medicine as an anti-inflammatory, analgesic and antiulcerogenic agent [33]. Likewise, extracts from stem bark and sap of *Staudtia kamerunensis* Warb. (Myristicaceae), an evergreen tree applied in traditional medicine in Central Africa to treat coughs, lung ailments, oedema, wounds and skin problems; exerted an increased activity against the pathogenic bacteria due to the synergism between pentacyclic triterpenoids and isoflavonoids [6]. The synergistic effects of various plant metabolites have become a key step in phytochemical studies, especially in the context of the growing problem with multi-drug-resistant bacterial and fungal strains. In such studies, the medicinal plants used for their antimicrobial and antifungal properties have been considered particularly inspiring, and the demonstrated therapeutic effects were comparable to those exerted by commercially available antibiotics [5,6]. 

The plants selected for the present study belong to various taxonomic taxa except for two *Cistus* species. As could be expected, *C. ladanifer* and *C. monspeliensis* displayed both similarities and differences in steroid and triterpenoid composition and content. The main profile of sterols and the types of triterpenoid skeletons were the same, the main difference refers to the amount of triterpenoids, particularly triterpenoid acids. However, this feature (i.e., the prevalence of triterpenoid acids in C. *monspeliensis*) cannot be considered universal, since the content of triterpenoids, classified as secondary (more aptly: specialized) metabolites, may differ significantly even between the plants of the same species growing in various habitats [31,32], and it can be modified under specific environmental conditions, e.g., biotic or abiotic stresses [34,35]. The study on *C. ladanifer* demonstrated a high intra-population variation of specialized metabolites, explained as a consequence of the interaction of the environmental variability of plant resources, genotypic differences among individuals, and herbivore or pathogen effects [36]. However, despite the significant differences in the content of individual compounds, their basic composition was similar. Likewise, regarding the triterpenoid and steroid profiles, the ability of biosynthesis of certain types of triterpenoid skeletons, or the presence of typical group of Δ^5^-phytosterols (or, on the contrary, Δ^7^-phytosterols), may have a significant chemotaxonomic value [37,38,39,40]. *Cistus* species are widely known and appreciated for their various pharmacological applications, ascribed mainly to various phenolic compounds including flavonoids and ellagitannins, as well as several classes of terpenoids, i.e., mono-, sesqui- and diterpenoids, particularly of labdane-type [41]; however, to our knowledge, steroid and triterpenoid contents have been analyzed in the present study for the first time. 

*E. arborea* has been pointed to be the most abundant source of both steroids and triterpenoids among the analyzed plants. Ericaceae plants can be generally considered rich sources of these compounds, as it was demonstrated previously for various *Vaccinium*, *Arbutus* and *Calluna* spp. [31,32,39,42]. Despite the abundance of steroids and triterpenoids in *E. arborea*, they have not been extensively analyzed so far, in contrast to other classes of compounds, as phenolics and essential oil constituents [22,23]. Only the presence of sitosterol was previously reported [22]. Meanwhile, the results obtained in this study revealed that *E. arborea* aerial parts (the mixture of flowers and leaves) contained relatively high amounts of the neutral triterpenoids and triterpenoid acids, notably α-amyrin and ursolic acid, combined with the presence of lupane-type compounds, including betulin. All these compounds exert various therapeutic and health-promoting activities, and have attracted great attention especially for their potent anticancer [43,44,45,46], antidiabetic [10] and antiviral [10] properties. Thus, *E. arborea* aerial parts can be recommended as a valuable source of bioactive triterpenoids. 

In contrast to *E. arborea*, the analysis of the *G. alypum* leaf extract revealed the relatively low content of both steroids and triterpenoids. According to ethnobotanical surveys, *G. alypum* is one of the most important medicinal plants in Algieria, traditionally used as an antidiabetic, laxative, stomachic, and purgative agent, mainly due to the occurrence of such compounds as flavonoids, tannins and iridoids [24,25,26]. However, the occurrence of triterpenoids, particularly betulin as well as ursolic acid, can be of interest in the context of the reported anti-inflammatory [47] and antituberculosis [24] activities, exerted by methanol and petroleum ether extracts, which might contain these low-polar compounds. Moreover, it was demonstrated that the best activity against *Mycobacterium tuberculosis* was obtained for the petroleum ether extract; therefore, the potential synergism of triterpenoids with other bioactive constituents cannot be ruled out. As was emphasized before, exploring the medicinal plants with antibacterial and antifungal activities can be particularly valuable in searching for the potential therapeutic agents against infections with multi-drug resistant strains. Recently, the ethanol extract from *Solanum torvum* Swartz (Solanaceae) fruit (a small tree cultivated in Africa and Asia, used in traditional medicine in the treatment of diabetes, hypertension, malaria and tuberculosis) containing triterpenoids, i.e., 3-oxo-friedelan-20α-oic, betulinic and oleanolic acids, has been demonstrated to be active against fluconazole-resistant clinical *Candida albicans* isolatates [15]. Additionally, the GC-MS analysis of *G. alypum* perform in the present study revealed the occurrence of significant content of policosanols, constituting a mixture of long-chain (C20 to C36) aliphatic primary alcohols exhibiting various beneficial effects on the human health. These compounds, originally isolated from sugar cane wax, beeswax and some vegetable oils, may be effective in the treatment of hypercholesterolemia, hypertension and metabolic syndrome [48]. It was proposed that medicinal plants, e.g., milk thistle *Silybum marianum* L. (Asteraceae) seeds, could also serve as a potential source of policosanols [49]. According to the present results, *G. alypum* leaves could be further explored as an alternative source of these compounds. 

The leaves of *P. lentiscus* were the second most abundant source of steroids and triterpenoids among the herbal material analyzed in the present study. It could be expected according to the reports on the triterpenoid content of this plant, concerning mainly the resin called mastic. The composition of triterpenoids in *P. lentiscus* mastic have been reported as particularly complex, comprising tetra- and pentacyclic triterpenoids as tirucallol, dammaradienone, β-amyrin, lupeol, oleanolic aldehyde, 28-norolean-12-en-3-one, as well as masticadienonic, isomasticadienonic, morolic, oleanonic, ursonic acids, and many other compounds [50,51,52]; moreover, the recent reports have been expanding this list [53]. *P. lentiscus* is also rich in other terpenoids present in essential oils, such as β-pinene, camphene, myrcene, limonene, β-caryophyllene and linalool [50]. The reported study on *P. lentiscus* fruit revealed the presence of phytosterols (sitosterol, campesterol, stigmasterols), several other steroids, and β-amyrin, additionally pointing to quantitative differences among the identified compounds according to the provenance [54]. In turn, the composition of phytoconstituents in leaves of *P. lentiscus* was very rarely investigated, mainly for phenolic compounds [55]. The analysis performed in the present study allowed to identify triterpenoids of several skeleton types, including 18-oleanane-type morolic and moronic acids, as well as oleanolic and 3-oxooleanolic acid. *P. lentiscus* leaves are cheaper, wider and readily available herbal material than mastic; therefore, according to the high content of phytosterols and triterpenoids, they might be also applied for further studies, e.g., on anti-inflammatory and anticancer properties. 

*R. alaternus*, as other representatives of the genus *Rhamnus* [56], is known to be rich in phenolic compounds, such as flavonoids, coumarins, tannins, anthraquinones and naphthalenes, that are considered responsible for antioxidant, antihyperlipidemic, antigenotoxic, antimutagenic, antimicrobial, and antiproliferative properties [29,30]. The analyzed contents of steroids and triterpenoids in *R. alaternus* leaves were not high; however, the triterpenoid profile was rather specific, consisting mainly of the neutral triterpenoids, including three alcohols: germanicol, and α- and β-amyrins. These compounds, similar to triterpenoid acids, exert antimicrobial, anti-inflammatory and other interesting biological activities [57], both as pure compounds, or synergistically in various mixtures. Plant-derived triterpenoids obtained from medicinal plants have been recently tested in terms of their antibacterial activity against multi-drug resistant strains, e.g., methicillin-resistant *Staphylococcus aureus* [6,58]. 

The comparison of the antioxidant potential of the analyzed extracts revealed that the highest free radical scavenging activity was exerted by *G. alypum* extract, which contained such antioxidants as α-tocopherol and α-tocopherylchinon. Generally, phytosterols and triterpenoids have not been regarded as primary compounds responsible for antioxidant properties exerted by plant extracts. Phytosterols have been considered the modest radical scavengers, acting mainly as stabilizers in the membranes [59]. Triterpenoids have been supposed to possess a higher antioxidant potential, particularly ascribed to triterpenoid acids [60]. Recently, the antioxidant activities exerted by triterpenoids occurring in various medicinal mushrooms have been intensively investigated [61]. It should be emphasized that the results concerning the antioxidant activity obtained in the present study cannot be compared with the results of DPPH tests reported for methanol or aqueous extracts for the analyzed plants, containing higher amounts of phenolic compounds, mainly responsible for antioxidant activity. 

The obtained results point to the importance of the selection of the solvent used for plant material extraction. The expected biological activities and pharmacological properties of phytosterols and triterpenoids can be demonstrated only by lipophilic extracts from the herbal material, because due to the low polarity of these compounds, they cannot be extracted by water. Therefore, the most popular folk medicine procedures as decoction and infusion, would not contain this important fraction of phytochemicals. Instead, dietary supplements or functional foods obtained with the use of lipophilic extracts from herbal material should be prepared in order to benefit from the wide spectrum of bioactivities exerted by phytosterols and triterpenoids.

According to the renewed interest in ethnopharmacology, traditional and alternative medicine, the current medical plants and herbal raw materials created a rapidly growing market. However, with the rising utilization of herbal products, there is a need for valuable guidelines on the selection, preparation, and application of herbal formulations in terms of their safety and efficacy. Further research on the potential side and toxic effects is also required. 

## 4. Materials and Methods

### 4.1. Plant Material

**Figure 4:**
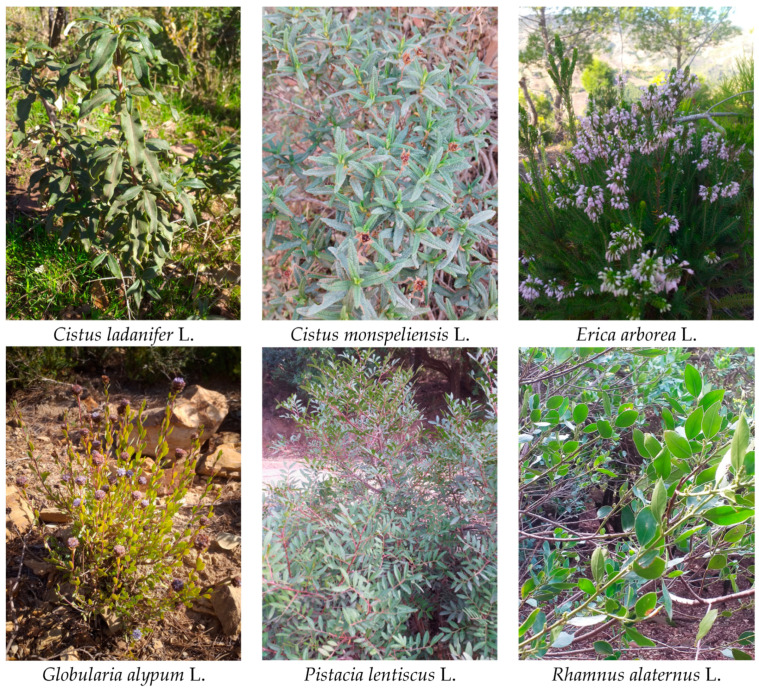
The selected medicinal plants in their natural habitat (the photographs taken by Dr. Leila Gadouche).

Plant samples: leaves of *Cistus ladanifer* L., *Cistus monspeliensis* L., *Globularia alypum* L., *Rhamnus alaternus* L. and an aerial part (flowers and leaves) of *Erica arborea* L. (Figure 4) were collected by Dr. Leila Gadouche in Beni Haoua (a commune in Chlef province in Algeria), which is a coastal forested mountainous region, characterized by a particular floristic composition [62]. The selected plants are widely applied in the local folk medicine, and they are known under many vernacular names: Oured for *C. ladanifer*; Om Alia, Tama itibt, Thouzelt, Tamechetibt for *C. monspeliensis*; Bouhaddad, Cheudef, Ariga, Aklelendj for *E. arborea*; Tasselgha, Chebra, Zerga for *G. alypum*; Derou, Tadist for *P. lentiscus*; M’liles, Aouid elkheir, Quaced for *R. alaternus*. 

The investigated plant species were identified by Dr. Fatima Belhacini, a botanist at the University of Ain Témouchent, and verified in the New Flora of Algeria and the southern desert regions of Quezel and Santa (1965) [63]. 

### 4.2. Extraction

Samples of air-dried plant material (three replicates each) were powdered, weighed and extracted in Soxhlet apparatus for 8 h with diethyl ether. The obtained extracts were evaporated and dried at 40 °C under reduced pressure on a rotary evaporator.

### 4.3. Fractionation of Diethyl Ether Extracts

Evaporated diethyl ether extracts were fractionated by adsorption preparative TLC on 20 cm × 20 cm glass plates, manually coated with silica gel 60H (Merck, Darmstadt, Germany). The solvent system chloroform: methanol 97:3 (*v*/*v*) was used to develop the plates. Two fractions were obtained: (i) free steroids and neutral triterpenoids (Rf of 0.3–0.9), and (ii) free triterpenoid acids (Rf 0.2–0.3) as described earlier [37]. The individual fractions were localized on plated by comparison with standards of oleanolic acid, sitosterol and α-amyrin, visualized by spraying the relevant part of the plate with 50% H_2_SO_4_, followed by heating with a hot-air stream. Fractions were eluted from the gel in diethyl ether. The fraction of free steroids and neutral triterpenoids was directly analyzed without derivatization using GC–MS (Agilent Technologies 7890A, Santa Clara, CA, USA) while the fraction of triterpenoid acids was methylated with diazomethane [31,32]. 

### 4.4. Derivatization of Triterpenoid Acids

Nitrosomethylurea (2.06 g) was added to a mixture of 20 mL of diethyl ether and 6 mL of 50% aqueous KOH. The organic layer was separated and used to dissolve the samples of triterpenoid acids (2 mL per sample). Reaction was held at 2 °C for 24 h.

### 4.5. Identification and Quantification of Steroids by Gas Chromatography-Mass Spectrometry

An Agilent Technologies 7890A gas chromatograph (GC–MS) (Perlan Technologies, Warszawa, Poland) equipped with a 5975C mass selective detector was used for qualitative and quantitative analyses. The column used was a 30 m × 0.25 mm i.d., 0.25-μm, HP-5MS UI (Agilent Technologies, Santa Clara, CA, USA). The carrier gas was helium, used at a flow rate of 1 mL/min. Analyzed samples were dissolved in the mixture of diethyl ether:methanol (5:1, *v*/*v*) and applied (in a volume of 1–4 μL) using 1:10 split injection. The separation was made with the temperature program: initial temperature of 160 °C held for 2 min, then increased to 280 °C at 5 °C/min, and the final temperature of 280 °C held for a further 44 min. The other employed parameters were as follows: inlet and FID (flame ionization detector) temperature 290 °C; MS transfer line temperature 275 °C; quadrupole temperature 150 °C; ion source temperature 230 °C; EI 70 eV; *m*/*z* range 33–500; FID gas (H2) flow 30 mL·min^−1^ (hydrogen generator HydroGen PH300, Peak Scientific, Inchinnan, UK); and airflow 400 mL·min^−1^. Individual compounds were identified by comparing their mass spectra with library data from Wiley 9th ED. and NIST 2008 Lib. SW Version 2010, or previously reported data, and by comparison of their retention times and corresponding mass spectra with those of authentic standards, where available. Quantitation was performed using an external standard method based on calibration curves determined for authentic standards of sitosterol, α-amyrin and oleanolic acid methyl ester, applied for each class of compounds (i.e., steroids, neutral triterpenoids and triterpenoid acids, respectively), as described earlier [31,37]. The correlation coefficient (r^2^) value for sitosterol was 0.998; and 0.999 for α-amyrin and oleanolic acid methyl ester. RSD values of peak areas used for the calibration curves were less than 4%. The LOD (limit of detection) values were 1.0 µg/mL for sitosterol, and 0.8 µg/mL for α-amyrin and oleanolic acid methyl ester. A 2.5 µg/mL LOQ (limit of quantification) was attained for both α-amyrin and oleanolic acid methyl ester, and 3.2 µg/mL for sitosterol [32]. 

### 4.6. Determination of Free Radical Scavenging Activity

The DPPH (2,2-diphenyl-1-picrylhydrazyl) method was used to measure free radical-scavenging activity as described earlier [64]. 2 mL of 0.1 mM DPPH in MeOH was added to 2 mL of MeOH containing different amounts of extract to produce final concentrations of 0 (control), 40, 80, 120, 240, 400 and 500 mg/mL. The absorbance at 517 nm was measured after 10 min. Triplicates of each sample were run and the mean values calculated. The scavenging of DPPH radical [%] was calculated according to the formula [(A0 − A1)/A0 × 100], where A0 is the absorbance of the control reaction and A1 is the absorbance of reactions containing analyzed extract.

### 4.7. Statistical Analysis of Data

Data are presented as the means ± standard deviation of three independent samples analyzed in triplicate. The data were subjected to one-way analysis of variance (ANOVA), and the differences between means were evaluated using Duncan’s multiple-range test. Statistical significance was considered to be obtained at *p* < 0.05.

## 5. Conclusions

GC-MS targeted profiling of triterpenoids and steroids in diethyl ether extracts obtained from selected medicinal plants of the Mediterranean region, i.e., *Cistus ladanifer*, *Cistus monspeliensis*, *Erica arborea*, *Globularia alypum*, *Pistacia lentiscus* and *Rhamnus alaternus* revealed the presence of typical phytosterols and pentacyclic triterpenoids of several types of carbon skeletons. Among phytosterols, sitosterol was predominating in all extracts, accompanied with campesterol and stigmasterol (except for *E. arborea* aerial parts, where stigmasterol was not detected). The highest phytosterol content was detected in *P. lentiscus* leaves (0.14 % d.w.), followed by *E. arborea* aerial parts (0.12% d.w.). The steroid ketones, sitostenone and tremulone were found in all extracts, whereas stigmasta-3,6-dione was detected only in *Cistus* spp, *R. alaternus* and *G. alypum*. 

The highest content of triterpenoids and their richest composition were determined in *E. arborea* aerial parts (approx. 5.6% d.w.). The neutral triterpenoids (α-amyrin, α-amyrenone, β-amyrin, β-amyrenone, betulin, erythrodiol, lupeol, lupenone, taraxasterol, oleanolic and ursolic aldehydes, uvaol) and triterpenoid acids (betulinic acid, maslinic acid, oleanolic acid, ursolic acid, olean-2,12-dien-28-oic and ursa-2,12-dien-28-oic acids, as well as 3-oxooleanolic and 3-oxoursolic acids) were identified in this plant. The most abundant compounds were α-amyrin (2.4% d.w.) and ursolic acid (1.5% d.w.) The second richest source of triterpenoids was *P. lentiscus* leaves (1% d.w.) containing additional morolic and moronic acids. The predominating compounds were α-amyrin (0.5% s.w.) and 3-oxooleanolic acid (0.32% d.w.).

To our knowledge, the detailed analysis of the profile of steroids and triterpenoids was performed on leaf lipophilic extracts of these plants, except for *Pistacia lentiscus*, for the first time in this study. The obtained results supplement the existing phytochemical characteristics of the investigated medicinal plants, pointing to the *E. arborea* aerial parts and *P. lentiscus* leaves as valuable resources of bioactive triterpenoids. 

## Figures and Tables

**Table 1:** Content of steroids and triterpenoids in *Cistus ladanifer* and *Cistus monspeliensis* leaves.

Compounds/Category	*Cistus ladanifer*	*Cistus monspeliensis*
	Content [µg/g d.w. ± SD]	
*sterols:*		
campesterol	40.24 ± 2.23 a	40.59 ± 2.55 a
sitosterol	571.97 ± 30.06 a	490.95 ±12.66 b
stigmasterol	22.60 ± 1.25 a	32.50 ± 2.46 b
*sum of sterols*	*634.81*	*564.04*
*steroid ketones:*		
sitostenone	24.75 ± 1.60 a	27.29 ± 1.69 a
stigmasta-3,6-dione	46.81 ± 1.81 a	58.64 ± 2.37 b
tremulone	22.51 ± 1.40 a	16.89 ± 1.64 b
*sum of steroid ketones*	*94.07*	*102.82*
***sum of steroids***	***728.88***	***666.86***
*neutral triterpenoids:*		
α-amyrin	280.80 ± 10.79 a	419.30 ± 20.71 b
β-amyrin	120.69 ± 3.33 a	370.40 ± 12.47 b
*sum of neutral triterpenoids*	*401.49*	*789.70*
*triterpenoid acids:*		
maslinic acid	31.82 ± 1.98 a	337.24 ± 15.98 b
maslinic acid methyl ester	n.d.	50.03 ± 2.49 a
oleanolic acid	34.61 ± 2.49 a	79.50 ± 11.48 b
3-oxooleanolic acid	19.10 ± 1.21 a	93.46 ± 13.62 b
ursolic acid	60.27 ± 2.58 a	8.49 ± 0.54 b
*sum of acids*	*145.80*	*568.72*
***sum of triterpenoids***	*547.29*	*1358.42*
**Total**	**1276.17**	**2025.28**

Results are referenced to leaf dry weight and expressed as the mean ± SD of three samples. Results not sharing a common letter (concerning the content of the same compound in all extracts) are significantly different (*p* < 0.05). n.d.—not detected.

**Table 2:** Content of steroids and triterpenoids in the aerial part of *Erica arborea*.

Compounds/Category	Content [µg/g d.w. ± SD]
*sterols:*	
campesterol	304.60 ± 10.52 b
sitosterol	846.15 ± 16.37 c
*sum of sterols*	*1150.75*
*steroid ketones:*	
sitostenone	50.49 ± 2.51 b
tremulone	82.73 ± 6.09 c
*sum of ketones*	*133.22*
***sum of steroids***	***1283.97***
*neutral triterpenoids:*	
α-amyrin/lupeol	23,809.86 ± 322.07 c
α-amyrenone/lupenone	502.10 ± 17.23 a
β-amyrin	2396.95 ± 87.87 c
β-amyrenone	618.19 ± 14.43 a
taraxasterol	75.52 ± 3.53 a
oleanolic aldehyde	74.46 ± 2.35 a
ursolic aldehyde	615.07 ± 18.70 a
betulin	204.78 ± 13.60 a
erythrodiol	38.53 ± 1.86 a
uvaol	158.09 ± 14.37 a
*sum of neutral triterpenoids*	*28,493.55*
*triterpenoid acids:*	
betulinic acid	573.12 ± 13.18 a
maslinic acid	641.61 ± 12.97 c
oleanolic acid	6022.89 ± 72.67 c
3-oxooleanolic acid	205.30 ± 15.97 c
olean-2,12-dien-28-oic acid	133.11 ± 7.15 a
ursolic acid	14,889.49 ± 129.99 c
3-oxoursolic acid	3570.32 ± 56.77 c
ursa-2,12-dien-28-oic acid	1007.38 ± 25.27 a
*sum of acids*	*27,043.22*
***sum of triterpenoids***	***55,536.77***
**Total**	**56,820.74**

Results are referenced to leaf dry weight and expressed as the mean ± SD of three samples. Results not sharing a common letter are significantly different (*p* < 0.05).

**Table 3:** Content of steroids and triterpenoids in *Globularia alypum* leaves.

Compounds/Category	Content [µg/g d.w. ± SD]
*sterols:*	
campesterol	24.63 ± 2.25 c
sitosterol	436.94 ± 20.46 d
stigmasterol	17.94 ± 2.09 c
*sum of sterols*	*479.51*
*steroid ketones:*	
sitostenone	62.61 ± 4.44 c
stigmasta-3,6-dione	95.31 ± 12.83 c
tremulone	53.34 ± 4.10 d
*sum of steroid ketones*	*211.26*
***sum of steroids***	***690.77***
*neutral triterpenoids:*	
α-amyrin/lupeol	52.06 ± 3.90 d
α-amyrenone/lupenone	26.77 ± 2.14 b
β-amyrin	36.35 ± 2.29 d
betulin	73.69 ± 10.64 b
***sum of neutral triterpenoids***	***188.87***
*triterpenoid acids:*	
oleanolic acid	47.98 ±3.87 d
ursolic acid	74.95 ± 5.36 d
***sum of acids***	***122.55***
***sum of triterpenoids***	***311.42***
**Total**	**1002.19**

Results are referenced to leaf dry weight and expressed as the mean ± SD of three samples. Results not sharing a common letter are significantly different (*p* < 0.05).

**Table 4:** Content of steroids and triterpenoids in *Pistacia lentiscus* leaves.

Compounds/Category	Content [µg/g d.w. ± SD]
*sterols:*	
campesterol	39.43 ± 2.18 a
sitosterol	1356.62 ± 60.63 e
stigmasterol	32.35 ± 2.57 b
*sum of sterols*	*1428.4*
*steroid ketones:*	
sitostenone	126.75 ± 15.16 d
tremulone	37.48 ± 2.23 d
*sum of ketones*	*164.23*
***sum of steroids***	***1592.63***
*neutral triterpenoids:*	
α-amyrin/lupeol	5121.09 ± 125.03 e
α-amyrenone/lupenone	606.64 ± 27.36 c
β-amyrin	307.68 ± 22.7 a
lupeol acetate	31.89 ± 3.36 a
oleanolic aldehyde	82.79 ± 11.11 a
ursolic aldehyde	96.57 ± 7.06 b
erythrodiol	28.41 ± 3.36 b
uvaol	33.26 ± 3.00 b
betulin	4.97 ± 0.14 c
*sum of neutral triterpenoids*	*6313.3*
*triterpenoid acids:*	
morolic acid	272.12 ± 14.17 a
moronic acid	118.27 ± 17.30 a
oleanolic acid	357.09 ± 17.39 b
3-oxooleanolic acid	3222.65 ± 74.40 d
*sum of acids*	*3970.13*
***sum of triterpenoids***	***10,283.43***
**Total**	**11,876.06**

Results are referenced to leaf dry weight and expressed as the mean ± SD of three samples. Results not sharing a common letter (*p* < 0.05).

**Table 5:** Content of steroids and triterpenoids in *Rhamnus alaternus* leaves.

Compounds/Category	Content [µg/g d.w. ± SD]
*sterols*	
campesterol	59.92 ± 3.79 d
sitosterol	309.10 ± 12.87 f
stigmasterol	66.49 ± 4.43 d
*sum of sterols*	*435.51*
*steroid ketones:*	
sitostenone	36.72 ± 1.88 e
stigmasta-3,6-dione	85.81 ± 5.55 d
tremulone	29.73 ± 1.69 a
*sum of ketones*	*152.26*
***sum of steroids***	***587.77***
*triterpenoids:*	
α-amyrin/lupeol	533.31 ± 20.86 b
β-amyrin	415.07 ± 25.32 b
germanicol	382.16 ± 20.73 a
oleanolic aldehyde	113.47 ± 11.80 b
ursolic aldehyde	203.64 ± 23.46 c
***sum of triterpenoids***	***1647.65***
**Total**	**2235.42**

Results are referenced to leaf dry weight and expressed as the mean ± SD of three samples. Results not sharing a common letter are significantly different (*p* < 0.05).

**Table 6:** Radical Scavenging Activity (RSA) of the analyzed extracts.

Extract	Radical Scavenging Activity (%)
*Cistus ladanifer*	27.8 ± 1.20
*Cistus monspelliensis*	69.9 ± 2.04
*Erica arborea*	77.3 ± 2.86
*Globularia alypum*	84.1 ± 3.05
*Pistacia lentiscus*	64.7 ± 2.12
*Rhamnus alaternus*	56.7 ± 1.81

Results are expressed as the mean of three samples.

## Data Availability

The data presented in this study are available in the article and Supplementary Materials.

## Supplementary Materials

The following supporting information can be downloaded at: https://www.mdpi.com/article/10.3390/molecules28020697/s1, Table S1. Retention times and characteristic ions of mass spectra of identified steroids and triterpenoids.

## References

[1] Aidi Wannes W., Saidani Tounsi M., Marzouk B. (2018). A review of Tunisian medicinal plants with anticancer activity. J. Complement. Integr. Med..

[2] Adigew M.G. (2022). Phytochemical analysis of some selected traditional medicinal plants in Ethiopia. Bull. Natl. Res. Cent..

[3] Sánchez M., González-Burgos E., Iglesias I., Lozano R., Gómez-Serranillos M.P. (2020). Current uses and knowledge of medicinal plants in the Autonomous Community of Madrid (Spain): A descriptive cross-sectional study. BMC Complement. Med. Ther..

[4] Fitzgerald M., Heinrich M., Booker A. (2020). Medicinal plant analysis: A historical and regional discussion of emergent complex techniques. Front. Pharmacol..

[5] Wrońska N., Szlaur M., Zawadzka K., Lisowska K. (2022). The synergistic effect of triterpenoids and flavonoids—New approaches for treating bacterial infections?. Molecules.

[6] Tonga J.L., Kamdem M.H.K., Pagna J.I.M., Fonkui T.Y., Tata C.M., Fotsing M.C.D., Nkengfack E.A., Mmutlane E.M., Ndinteh D.T. (2022). Antibacterial activity of flavonoids and triterpenoids isolated from the stem bark and sap of *Staudtia kamerunensis* Warb. (Myristicaceae). Arab. J. Chem..

[7] Fang S., Belwal T., Li L., Limwachiranon J., Liu X., Luo Z. (2020). Phytosterols and their derivatives: Potential health-promoting uses against lipid metabolism and associated diseases, mechanism and safety issues. Compr. Rev. Food Sci. Food Saf..

[8] Suryamani, Sindhu R., Singh I., Cazarin C.B.B., Pastore G.M., Bicas J.L., Morostica M.R. (2022). Phytosterols: Physiological functions and therapeutic applications. Bioactive Food Components Activity in Mechanistic Approach.

[9] Li X., Xin Y., Mo Y., Marozik P., He T., Guo H. (2022). The bioavailability and biological activities of phytosterols as modulators of cholesterol metabolism. Molecules.

[10] Alqahtani A., Hamid K., Kam A., Wong K.H., Adelhak Z., Razmovski-Naumovski V., Chan K., Li K.M., Groundwater P.W., Li G.Q. (2013). The pentacyclic triterpenoids in herbal medicines and their pharmacological activities in diabetes and diabetic complications. Curr. Med. Chem..

[11] Paduch R., Kandefer-Szerszeń M. (2014). Antitumor and antiviral activity of pentacyclic triterpenes. Mini-Rev. Org. Chem..

[12] Xiao S., Tian Z., Wang Y., Si L., Zhang L., Zhou D. (2018). Recent progress in the antiviral activity and mechanism study of pentacyclic triterpenoids and their derivatives. Med. Res. Rev..

[13] Sandeep, Ghosh S., Atta-ur-Rahman (2020). Triterpenoids: Structural diversity, biosynthetic pathways and bioactivity. Studies in Natural Products Chemistry.

[14] Tolufashe G.F., Lawal M.M., Govender K.K., Shode F.O., Singh T. (2021). Exploring the bioactivity of pentacyclic triterpenoids as potential antimycobacterial nutraceutics: Insights through comparative biomolecular modeling. J. Mol. Graph. Model..

[15] Harley B.K., Neglo D., Tawiah P., Pipim M.A., Mireku-Gyimah N.A., Tettey C.O., Amengor C.D., Fleisher T.C., Waikhom S.D. (2021). Bioactive triterpenoids from *Solanum torvum* fruits with antifungal, resistance modulatory and antibiofilm formation activities against fluconazole-resistant *Candida albicans* strains. PLoS ONE.

[16] Cocco E., Maccioni D., Sanjust E., Falconieri D., Farris E., Maxia A. (2022). Ethnopharmacobotany and diversity of Mediterranean endemic plants in Marmilla subregion, Sardinia, Italy. Plants.

[17] Papaefthimiou D., Papanikolaou A., Falaza V., Givanoudi S., Kostas S., Kanellis A.K. (2014). Genus Cistus: A model for exploring lab dane-type diterpenes’ biosynthesis and a natural source of high value products with biological, aromatic, and pharmacological properties. Front. Chem..

[18] El Karkouri J., Bouhrim M., Al Kamaly O.M., Mechchate H., Kchibale A., Adadi I., Amine S., Alaoui Ismaili S., Zair T. (2021). Chemical composition, antibacterial and antifungal activity of the essential oil from *Cistus ladanifer* L. Plants.

[19] Sayah K., Marmouzi I., Mrabti H.N., Cherrah Y., El Abbes Faouzi M. (2017). Antioxidant activity and inhibitory potential of *Cistus salviifolius* (L.) and *Cistus monspeliensis* (L.) aerial parts extracts against key enzymes linked to hyperglycemia. BioMed Res. Intl..

[20] Haida S., Bakkouche K., Kribii A.R., Kribii A. (2021). Chemical composition of essential oil, phenolic compounds, and antioxidant activity of *Cistus monspeliensis* from Northern Morocco. Biochem. Res. Int..

[21] Dias P., Falé P.L., Martins A., Rauter A.P. (2015). Digestibility and bioavailability of the active components of *Erica australis* L. aqueous extracts and their therapeutic potential as acetylcholinesterase inhibitors. Evid.-Based Complement. Altern. Med..

[22] Nazemiyeh H., Bahadori F., Delazar A., Ay M., Topçu G., Nahar L., Majinda R.R.T., Sarker S.D. (2008). Antioxidant phenolic compounds from the leaves of *Erica arborea* (Ericaceae). Nat. Prod. Res..

[23] Bessah R., Benyoussef E.-H. (2014). Essential oil composition of *Erica arborea* L. leaves from Algeria. J. Essent. Oil-Bear. Plants.

[24] Khlifi D., Hamdi M., El Hayouni A., Cazaux S., Souchard J.-P., Couderc F., Bouajila J. (2011). Global chemical composition and antioxidant and anti-tuberculosis activities of various extracts of *Globularia alypum* L. (Globulariaceae) leaves. Molecules.

[25] Asraoui F., Kounnoun A., Cadi H.E., Cacciola F., Majdoub Y.O.E., Alibrando F., Mandolfino F., Dugo P., Mondello L., Louajri A. (2021). Phytochemical investigation and antioxidant activity of *Globularia alypum* L.. Molecules.

[26] Friščić M., Petlevski R., Kosalec I., Madunić J., Matulić M., Bucar F., Hazler Pilepić K., Maleš Ž. (2022). *Globularia alypum* L. and related species: LC-MS profiles and antidiabetic, antioxidant, anti-inflammatory, antibacterial and anticancer potential. Pharmaceuticals.

[27] Bozorgi M., Memariani Z., Mobli M., Surmaghi M.H.S., Shams-Ardekani M.R., Rahimi R. (2013). Five *Pistacia* species (*P. vera*, *P. atlantica*, *P. terebinthus*, *P. khinjuk*, and *P. lentiscus*): A review of their traditional uses, phytochemistry, and pharmacology. Sci. World J..

[28] Ghzaiel I., Zarrouk A., Nury T., Libergoli M., Florio F., Hammouda S., Ménétrier F., Avoscan L., Yammine A., Samadi M. (2021). Antioxidant properties and cytoprotective effect of *Pistacia lentiscus* L. seed oil against 7β-hydroxycholesterol-induced toxicity in C2C12 myoblasts: Reduction in oxidative stress, mitochondrial and peroxisomal dysfunctions and attenuation of cell death. Antioxidants.

[29] Nekkaa A., Benaissa A., Mutelet F., Canabady-Rochelle L. (2021). *Rhamnus alaternus* plant: Extraction of bioactive fractions and evaluation of their pharmacological and phytochemical properties. Antioxidants.

[30] Gadouche L., Zerrouki K., Zidane A., Ababou A., Bachir Elazaar I., Merabet D., Henniche W., Ikhlef S. (2022). Genoprotective, antimutagenic, and antioxidant effect of methanolic leaf extract of *Rhamnus alaternus* L. from the Bissa mountains in Algeria. Foods Raw Mater..

[31] Szakiel A., Pączkowski C., Koivuniemi H., Huttunen S. (2012). Comparison of the triterpenoid content of berries and leaves of lingonberry *Vaccinium vitis-idaea* from Finland and Poland. J. Agric. Food Chem..

[32] Szakiel A., Pączkowski C., Huttunen S. (2012). Triterpenoid content of berries and leaves of bilberry *Vaccinium myrtillus* from Finland and Poland. J. Agric. Food Chem..

[33] Dos Santos V.A., Dos Santos D.P., Castro-Gamboa I., Zanoni M.V., Furlan M. (2010). Evaluation of antioxidant capacity and synergistic associations of quinonemethide triterpenes and phenolic substances from *Maytenus ilicifolia* (Celastraceae). Molecules.

[34] Rogowska A., Pączkowski C., Szakiel A. (2022). Modulation of steroid and triterpenoid metabolism in *Calendula officinalis* plants and hairy root cultures exposed to cadmium stress. Int. J. Mol. Sci..

[35] Rogowska A., Stpiczyńska M., Pączkowski C., Szakiel A. (2022). The influence of exogenous jasmonic acid on the biosynthesis of steroids and triterpenoids in Calendula officinalis plants and hairy root culture. Int. J. Mol. Sci..

[36] Masa C.V., Gallego J.C.A., Lobón N.C., Díaz T.S. (2016). Intra-population variation of secondary metabolites in *Cistus ladanifer* L. Molecules.

[37] Szakiel A., Grabarczyk M., Pączkowski C., Mieczkowski A. (2017). Comparison of the profiles of non-glycosylated triterpenoids from leaves of plants of selected species of genus *Dioscorea*. Phyt. Lett..

[38] Rogowska A., Styczyński M., Pączkowski C., Szakiel A., Pinheiro de Carvalho M.Â.A. (2019). GC-MS analysis of steroids and triterpenoids occurring in leaves and tubers of *Tamus edulis* Lowe. Phyt. Lett..

[39] Dashbaldan S., Becker R., Pączkowski C., Szakiel A. (2019). Various patterns of composition and accumulation of steroids and triterpenoids in cuticular waxes from screened Ericaceae and Caprifoliaceae berries during fruit development. Molecules.

[40] Dashbaldan S., Pączkowski C., Szakiel A. (2020). Variations in triterpenoid deposition in cuticular waxes during development and maturation of selected fruits of Rosaceae family. Int. J. Mol. Sci..

[41] Zalegh I., Akssira M., Bourhia M., Mellouki F., Rhallabi N., Salamatullah A.M., Alkaltham M.S., Khalil Alyahya H., Mhand R.A. (2021). A Review on Cistus sp.: Phytochemical and antimicrobial activities. Plants.

[42] Szakiel A., Niżyński B., Pączkowski C. (2013). Triterpenoid profile of flower and leaf cuticular waxes of heather *Calluna vulgaris*. Nat. Prod. Res..

[43] Shangmugam M.K., Nguyen A.H., Kumar A.P., Tan B.K.H., Sethi G. (2012). Targeted inhibition of tumor proliferation, survival, and metastasis by pentacyclic triterpeoids: Potential role in prevention and therapy of cancer. Cancer Lett..

[44] Woźniak Ł., Skąpska S., Marszałek K. (2015). Ursolic acid—A pentacyclic triterpenoid with a wide spectrum of pharmacological activities. Molecules.

[45] Ghonte M.H., Jamkhande P.G. (2019). Role of pentacyclic triterpenoids in chemoprevention and anticancer treatment: An overview on targets and underlying mechanisms. J. Pharmacopunct..

[46] Sharma N., Palia P., Chaudhary A., Shalini, Verma K., Kumar I. (2020). A rewiew on pharmacological activities of lupeol and its triterpene derivatives. J. Drug Deliv. Ther..

[47] Boutemak K., Safta B., Ayachi N. (2015). Study of the antiinflammatory activity of flavonic extract of *Globularia alypum* L.. Acta Phys. Pol. A.

[48] Kim S.-J., Yadav D., Park H.-J., Kim J.-R., Cho K.-H. (2018). 2018. Long-Term consumption of cuban policosanol lowers central and brachial blood pressure and improves lipid profile with enhancement of lipoprotein properties in healthy Korean participants. Front. Physiol..

[49] Harrabi S., Ferchichi A., Bacheli A., Fellah H. (2018). Policosanol composition, antioxidant and anti-arthritic activities of milk thistle (*Silybum marianum* L.) oil at different seed maturity stages. Lipids Health Dis..

[50] Hadjimbei E., Botsaris G., Goulas V., Gekas V. (2015). Health-promoting effects of *Pistacia* resins: Recent advances, challenges, and potential applications in the food industry. Food Rev. Int..

[51] Yu Y.-H., Feng Y.-P., Liu W., Yuan T. (2022). Diverse triterpenoids from mastic produced by Pistacia lentiscus and their antiinflammtory activities. Chem. Biodivers..

[52] Liu W., Gao J., Li M., Aisa H.A., Yuan T. (2022). Tirucallane triterpenoids from the mastic (*Pistacia lentiscus*) and their anti-inflammatory and cytotoxic activities. Phytochemistry.

[53] An X., Wang J., Yu X., Wu H., Liu W. (2022). Two new polypodane-type bicyclic triterpenoids from mastic. Open Chem..

[54] Trabelsi H., Sakouhi F., Renaud J., Villeneuve P., Khouja M.L., Mayer P., Boukhchina S. (2012). Fatty acids, 4-desmethylsterols, and triterpene alcohols from Tunisian lentisc (*Pistacia lentiscus*) fruits. Eur. J. Lipid Sci. Technol..

[55] Qabaha K., Ras S.A., Abbadi J., Al-Rimawi F. (2016). Anti-inflammatory activity of *Eucalyptus* spp. and *Pistacia lentiscus* leaf extracts. Afr. J. Tradit. Complement. Altern. Med..

[56] Chen G., Li V., Soler F., Guo M. (2016). Analysis of flavonoids in *Rhamnus davrica* and its antiproliferative activities. Molecules.

[57] Vázquez L.H., Palazon J., Navarro-Ocańa A., Rao V. (2022). The pentacyclic triterpenes α, β-amyrins: A review of sources and biological activities. Phytochemicals—A Global Perspective of Their Role in Nutrition and Health.

[58] Guefack M.-G.F., Ngangoue M.O., Mbaveng A.T., Nayim P., Kuete J.R.N., Carine Ngafo M.N., Chi G.F., Ngameni B., Ngadjui B.T., Kuete V. (2022). Antibacterial and antibiotic-potentiation activity of the constituents from aerial part of Donella welwitshii (Sapotaceae) against multidrug resistant phenotypes. BMC Complement. Med. Ther..

[59] Yoshida Y., Niki E. (2003). Antioxidant effects of phytosterol and its component. J. Nutr. Sci. Vitaminol..

[60] Fu Y., Zhang Y., Zhang R. (2021). Purification and antioxidant properties of triterpenoid acids from blackened jujube (*Ziciphus jujuba* Mill.) by macroporous resin. Food Sci. Nutr..

[61] Wang C., Liu X., Lian C., Ke J., Lin J. (2019). Triterpenes and aromatic meroterpenoids with antioxidant activity and neuroprotective effects from *Ganoderma lucidum*. Molecules.

[62] Ababou A., Chouieb M., Saidi D., Bouthiba A., Mederbal K. (2017). Analyse statistique de la diversité floristique dans la région de Beni-Haoua, Chlef, Algérie. NATEC.

[63] Quezel P., Santa S. (1963). New Flora of Algeria and Southern Desert Regions.

[64] Szakiel A., Voutquenne-Nazabadioko L., Henry M. (2011). Isolation and biological activities of lyoniside from rhizomes and stems of *Vaccinium myrtillus*. Phyt. Lett..

