# Supplementary material for: Triterpenoid and Steroid Content of Lipophilic Extracts of Selected Medicinal Plants of the Mediterranean Region
Source: Molecules. 2023 Jan 10;28(2):697. doi: 10.3390/molecules28020697 (PMC9866667; doi:10.3390/molecules28020697)
Supplement: Supplementary file 1 [file molecules-28-00697-s001.zip › molecules-2101793-supplementary.pdf]

Triterpenoid and Steroid Content of Lipophilic Extracts of Selected Medicinal Plants of the Mediterranean Region

Table S1. Retention times and characteristic ions of mass spectra of identified steroids and triterpenoids

Retention time	Compound	Mass spectrum *m*/*z* (relative intensity)
34.6	Campesterol	400 (30), 107 (51), 105 (55), 95 (49), 83 (45), 81 (64), 71 (62), 57 (77), 55 (77), 43 (100), 41 (52)
35.5	Stigmasterol	412 (36), 145 (64), 107 (52), 95 (100), 83 (66), 81 (90), 78 (60), 69 (67), 67 (85), 55 (69)
37.5	Sitosterol	414 (29), 145 (54), 107 (59), 105 (60), 95 (54), 91 (49), 81 (57), 57 (68), 55 (70), 43 (100), 41 (44)
37.8	β-Amyrenone	424 (11), 219 (18), 218 (100), 205 (13), 203 (55), 189 (14), 109 (12), 95 (16), 81 (12), 69 (14), 55 (15)
38.2	Germanicol	426 (1), 204 (100), 177 (85), 189 (75), 95 (58), 55 (46), 205 (44), 81 (42), 109 (40), 69 (39), 107 (37)
38.5	β-Amyrin	426 (27), 219 (18), 218 (100), 203 (49), 189 (17), 135 (11), 109 (13), 105 (12), 95 (15), 81 (18), 69 (14)
39.5	α-Amyrenone	424 (12), 219 (19), 218 (100), 203 (24), 189 (16), 135 (19), 133 (18), 122 (18), 119 (17), 95 (16), 55 (18)
39.4	α-Amyrin/	426 (4), 218 (100), 203 (20), 189 (36), 135 (35), 121 (32), 109 (32), 107 34), 95 (40), 81 (33), 55 (31)
	Lupeol	426 (18), 207 (67), 189 (90), 135 (83), 121 (80) 109 (85), 121 (80), 95 (100), 93 (87), 81 (86),
40.7	Tremulone (stigmasta-3,5-dien-7-one)	410 (32), 187 (27), 174 (100), 161 (37), 159 (26), 91 (28), 57 (28), 55 (37), 43 (44), 41 (28)
41.9	Sitostenone	440 (5), 121 (60), 119 (55), 109 (62), 107 (76), 105 (57), 95 (98), 93 (64), 81 (72), 69 (99), 55 (100)
43.5	Taraxasterol	426 (14), 207 (57), 189 (100), 135 (51), 121 (74), 109 (57), 107 (62), 95 (70), 93 (47), 81 (48), 67 (43)
44.0	Lupeol acetate	468 (8), 189 (100), 135 (63), 121 (76), 109 (71), 107 (78), 95 (77), 93 (80), 81 (68), 69 (53)
48.0	Oleanolic aldehyde	440 (2), 232 (28), 207 (20), 204 (39), 203 (100), 189 (29), 105 (18), 81 (19), 69 (20), 55 (29)
49.9	Stigmastane-3.6-dione	428 (25), 135 (61), 107 (74), 98 (63), 95 (67), 79 (62), 69 (86), 57 (67), 55 (100), 43 (77), 41 (71)
50.9	Ursolic aldehyde	440 (1), 207 (26), 204 (23), 203 (100), 133 (42), 119 (18), 105 (18), 95 (18), 81 (18), 55 (18), 43 (20)

52.7	Erythrodiol	442 (1), 204 (17), 203 (100), 133 (7), 119 (9), 105 (8), 95 (9), 93 (8), 81 (8), 69 (9), 55 (8)
55.7	Uvaol	442 (1), 207 (13), 204 (17), 203 (100), 133 (33), 119 (13), 105 (11), 95 (12), 81 (10), 69 (10), 55 (11)
57.2	Betulin	442 (8), 203 (100), 189 (77), 133 (66), 121 (55), 107 (57), 105 (49), 95 (56), 93 (54), 81 (67)
Acids analyzed after methylation:		
42.0	Olean-2,12-dien-28-oic acid methyl ester	452(11), 425 (9), 263 (11), 262 (61), 221 (14), 203 (100), 190 (15), 189 (22), 133 (14), 119 (12)
44.7	Ursa-2,12-dien-28-oic acid methyl ester	452 (12), 425 (9), 263 (20), 262 (100),221 (27), 203 (79), 190 (18), 189 (27), 133 (58), 119 (23)
44.7	Moronic acid methyl ester	468 (17), 248 (35), 205 (15), 203 (30), 190 (24), 189 (100), 187 (26), 133 (17), 121 (28), 119 (28)
46.0	3-Oxo-olean-12-en-28-oic acid methyl ester	468 (6), 262 (32), 204 (17), 203 (100), 202 (21), 189 (29),133 (17), 119 (14), 105 (12), 55 (12)
46.2	Morolic acid methyl ester	470 (80), 263 (17), 262 (86), 249 (50), 247 (50), 208 (49), 207 (20), 204 (16), 203 (100), 202 (26)
46.7	Oleanolic acid methyl ester	470 (1), 262 (48), 207 (13), 204 (16), 203 (100), 202 (21), 189 (22), 133 (17), 119 (13), 105 (14)
47.1	Betulinic acid methyl ester	470 (5), 207 (41), 203 (38), 189 (100), 175 (40), 119 (41), 107 (38), 105 (37), 95 (37), 93 (38)
47.5	3-Oxo-urs-12-en-28-oic acid methyl ester	468 (3), 263 (21), 262 (96), 249 (20), 204 (17), 203 (100), 189 (29), 133 (79), 119 (30), 105 (19)
49.7	Ursolic acid methyl ester	470 (1), 263 (20), 262 (100), 207 (32), 203 (93), 189 (29), 133 (76), 119 (34), 105 (21), 95 (18)
61.0	Maslinic acid methyl ester	486 (2), 263 (10), 262 (53), 204 (17), 203 (100), 202 (20), 189 (20), 133 (16), 119 (13), 105 (12), 69 (10)
